# An unusual cause of small bowel obstruction due to an ingested mango seed: a case report

**DOI:** 10.1186/s13104-017-2875-3

**Published:** 2017-11-02

**Authors:** A. R. Fernando, R. Bulathsinghela, D. N. Samarasekera

**Affiliations:** 1 Professorial Surgical Unit, National Hospital of Sri Lanka, Colombo, 00700 Sri Lanka; 2 Professorial Surgical Unit, Faculty of Medicine, University of Colombo, Colombo, 00700 Sri Lanka

**Keywords:** Intestinal obstruction, Laparotomy, Mango seed

## Abstract

**Background:**

Intestinal obstruction can occur due to multiple aetiologies. Intestinal obstruction due to phytobezoar have been reported. However, intestinal obstruction due to a mango seed has not been reported. Therefore, accidental ingestion of a mango seed is rare, and for an ingested mango seed to cause intestinal obstruction is rarer.

**Case presentation:**

This case report is of a male who accidentally ingested a mango seed and presented with intestinal obstruction. The obstruction was at the terminal ileum. It required laparotomy for retrieval.

**Conclusion:**

It is extremely rare for a mango seed to cause intestinal obstruction. Hence, diagnosis requires a high degree of clinical suspicion. Instead of laparotomy, studies have demonstrated the use of laparoscopy for removal of ingested seeds.

## Background

Small bowel obstruction can occur due to extramural, mural or intraluminal causes, and adhesions account for more than 60%, followed by Crohn’s disease and malignancy. Foreign bodies in the gastrointestinal tract can get obstructed depending on their shape and consistency. Frequent sites of obstruction include the pylorus, ‘C’ loop of the duodenum and the ileocaecal junction [1].

Conventional management of acute intestinal obstruction is to perform a laparotomy to relieve the obstruction. However, laparoscopic management of intestinal obstruction due to foreign bodies are also described in the literature [2].

## Case presentation

A 40 year old male presented to the surgical casualty ward with central colicky abdominal pain, which progressively got worse over 4 days. This was following an accidental ingestion of a single mango seed while being under the influence of alcohol.

He developed abdominal distension, absolute constipation and associated bilious vomiting on the 4th day just prior to admission to hospital. He had no comorbidities and no previous abdominal surgeries to suggest adhesive obstruction.

On examination, patient appeared to be dehydrated, ill looking and in pain. Pulse rate was 95 beats/min and his blood pressure was 130/80 mmHg. His abdomen was distended with no features of peritonitis. Auscultation revealed increased bowel sounds. Digital rectal examination revealed an empty rectum.

**Fig. 1:**
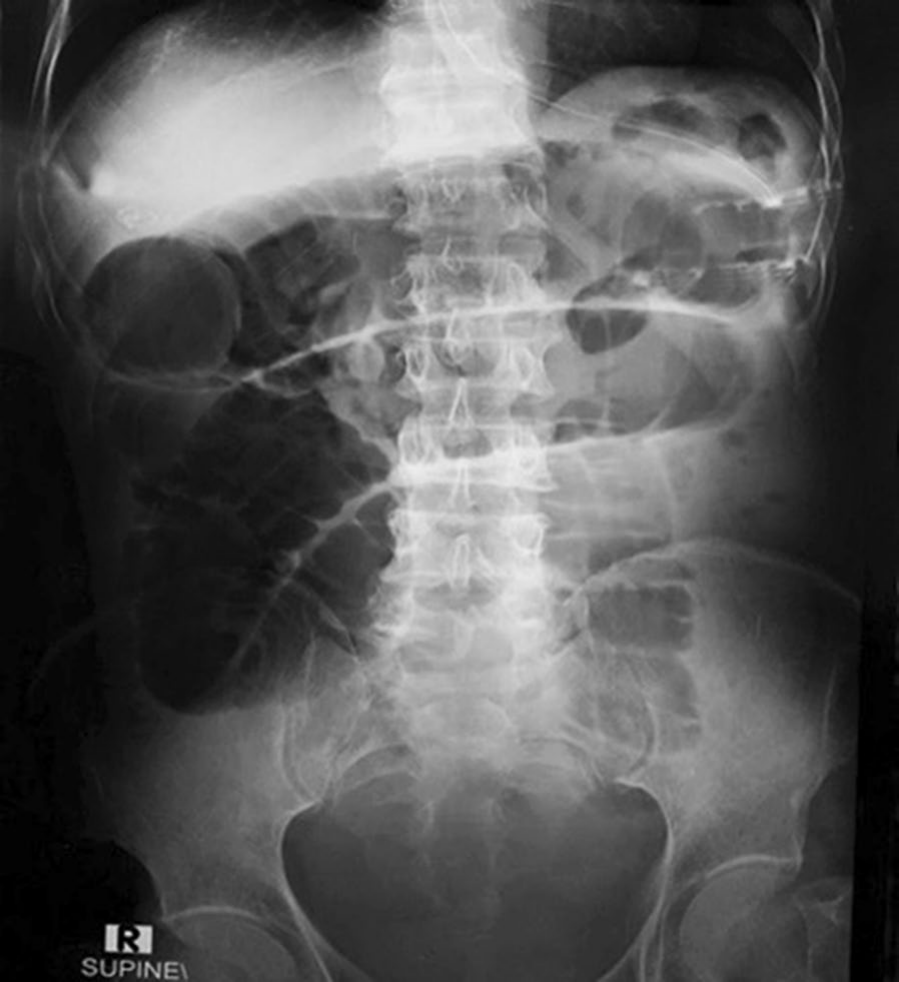
Supine abdominal X-ray shows multiple dilated small bowel loops with absent rectal gas shadows

**Fig. 2:**
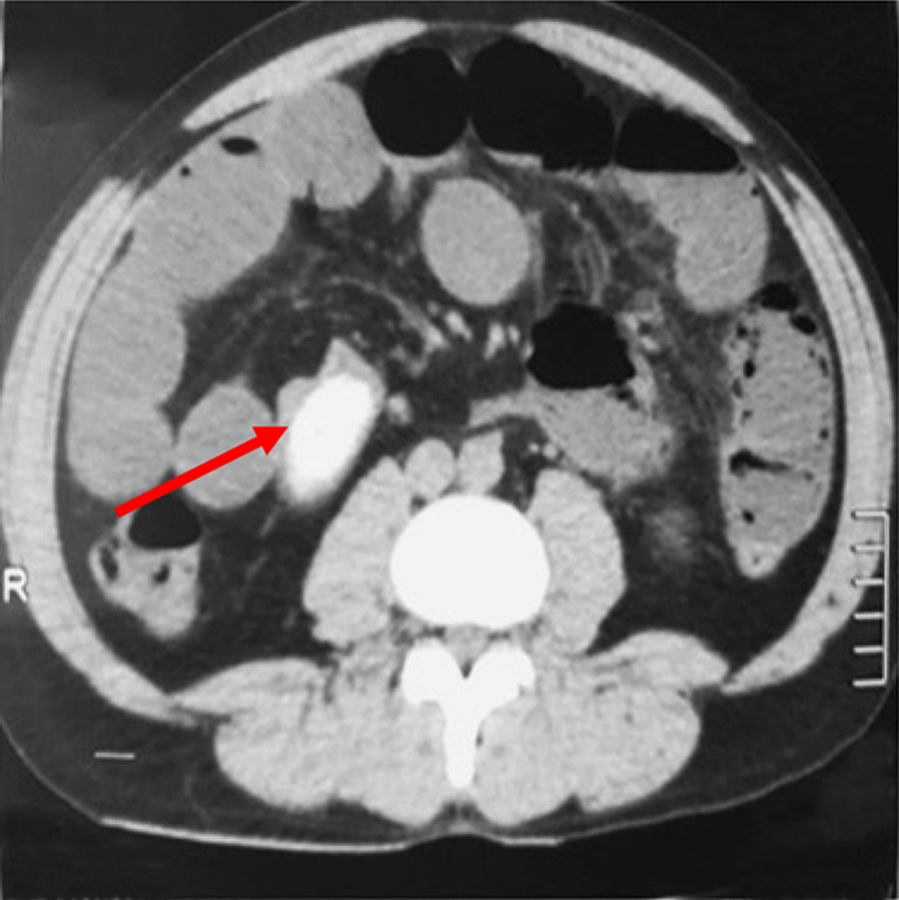
Axial section of NCCT abdomen showing dilated bowel loops with the mango seed (arrow)

**Fig. 3:**
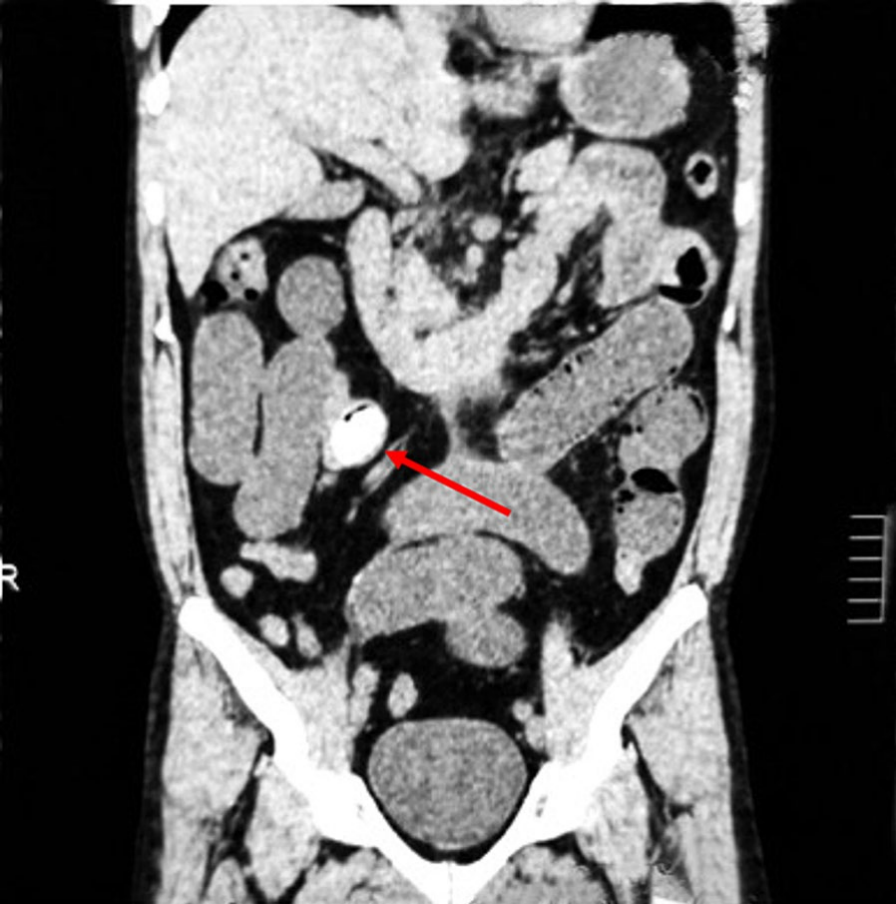
Coronal section of NCCT abdomen showing dilated bowel loops with the mango seed (arrow)

Abdominal supine X-ray showed multiple dilated jejunal and ileal loops with absent rectal gas shadows (Fig. 1). Non-contrast CT (computerized tomography) scan of abdomen was performed which revealed a foreign body located in the distal ileum with complete obstruction of the lumen (Figs. 2, 3).

**Fig. 4:**
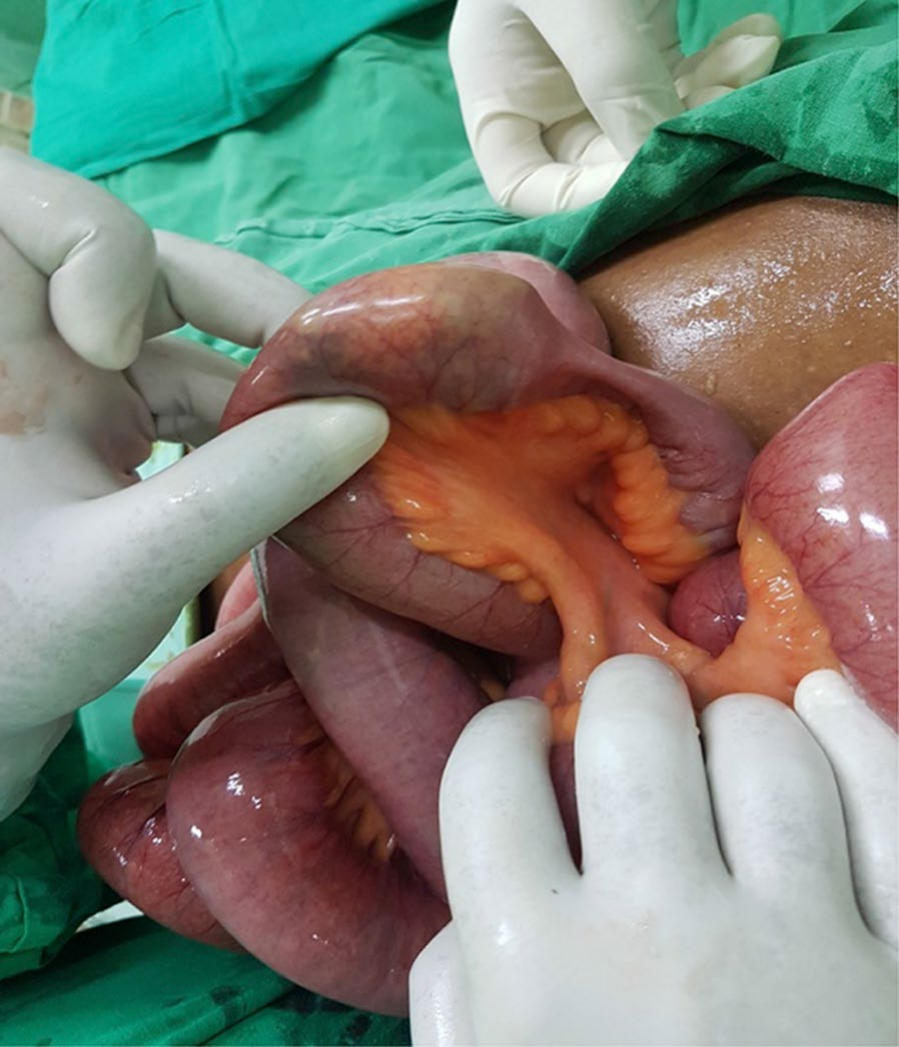
Intraoperative findings with distended proximal small bowel loops and the mango seed causing obstruction

Therefore, a midline laparotomy was performed under general anaesthesia. Distended small bowel loops noted with a mango seed completely obstructing the lumen 20 cm from the ileocaecal junction. There was complete collapse of the bowel loops distal to the obstruction (Fig. 4). The mango seed could not be milked beyond the obstruction to the colon, and therefore a longitudinal enterotomy was done and the foreign body (mango seed) was retrieved. Bowel decompression was also done through the same enterotomy. The enterotomy was closed with serosubmucosal-interrupted stitches using 3–0 polyglactin sutures.

The postoperative period was uneventful and the patient was discharged on the 4th postoperative day.

## Discussion and conclusions

Sri Lanka is a tropical country with an ample supply of fruits and vegetables. Even though per capita fruit consumption remains low [3], ingestion of mango remains common. However, mango seeds are usually discarded and not consumed. In this particular instance since the patient was under the influence of alcohol, accidental ingestion occurred.

A mango seed causing intestinal obstruction has not been reported. However, there are reports of phytobezoar causing intestinal obstruction [4]. Phytobezoar is a type of trapped indigestible plant material in the human gastrointestinal tract. This includes fibres, seeds and skin of plants.

In this particular case report, there wasn’t sufficient time or indigestible material to cause intestinal obstruction due to a formed phytobezoar, instead it was a mango seed causing intestinal obstruction.

Mango seed is a foreign body. Foreign bodies usually get obstructed within the oesophagus, pylorus, duodenal curve and the ileocaecal junction. In the oesophagus, it may get impacted at the upper oesophageal sphincter (15–17 cm from incisors), aortic arch impression (22–25 cm from incisors) or at the lower oesophageal sphincter (35–40 cm from incisors) [1].

Foreign body that reaches the stomach has an 80–90% chance of passage. And almost all pass through, once it reaches the small bowel negotiating the pylorus and duodenal curve. It rarely gets impacted at the ileocaecal valve. Time taken for natural passage is about 4–6 days and rarely up to 4 weeks.

The likelihood of an ingested foreign body to pass out through the anus depends on the size and shape. Chance of passage is more when the width of the foreign body is less than 2.5 cm which passes through the pylorus and when the length of the foreign body is less than 6 cm, which helps to negotiate the acute angles of the duodenal curve.

This particular mango seed was 3 cm in width and 5 cm in length, but despite the fact that it negotiated the pylorus and duodenal curve it got impacted at the terminal ileum.

Plain radiograph of the abdomen is the most valuable initial diagnostic test in acute small bowel obstruction (SBO). It is easily available. This imaging method gives information diagnostic of SBO in 50–60% of cases and provide enough information needed for clinical decision making [5].

Computed tomography (CT scan) is emerging as a valuable tool in the management of bowel obstruction. Sensitivity for diagnosis is high as 95%. It confirms the diagnosis, differentiates between mechanical and functional obstruction, and provides information about cause and site of obstruction. In this particular case the mango seed was clearly seen on the NCCT (non-contrast computerized tomography) abdomen at terminal ileum [6].

In Sri Lanka, laparoscopic approach to remove foreign body is rare, as it requires expertise. Therefore, frequently in Sri Lanka emergency laparotomy is performed to retrieve foreign bodies causing obstruction. Since obstruction by a mango seed is even rarer, laparotomy was performed to retrieve the mango seed. However, laparoscopic approach is also an option for retrieval of mango seed [7].

## Abbreviations

CT: computerized tomography
SBO: small bowel obstruction
NCCT: non-contrast computerized tomography
